# Iatrogenic injury of the urinary tract during salvage procedures for pelvic sepsis: Experience of a national referral centre

**DOI:** 10.1111/codi.16221

**Published:** 2022-07-11

**Authors:** Johanna J. Joosten, Sarah Sharabiany, Gijsbert D. Musters, Harrie P. Beerlage, Pieter J. Tanis, Wilhelmus A. Bemelman, Roel Hompes

**Affiliations:** ^1^ Department of Surgery, Amsterdam UMC University of Amsterdam Amsterdam The Netherlands; ^2^ Department of Urology, Amsterdam UMC University of Amsterdam Amsterdam The Netherlands; ^3^ Department of Surgery, Amsterdam UMC, Cancer Centre Amsterdam University of Amsterdam Amsterdam The Netherlands; ^4^ Department of Surgical Oncology and Gastrointestinal Surgery Rotterdam The Netherlands

**Keywords:** anastomotic leakage, pelvic sepsis, rectal cancer, salvage surgery, urinary complications

## Abstract

**Aim:**

This study aimed to determine the incidence, consequences and outcomes of iatrogenic urinary tract injury (IUI) during salvage surgery for pelvic sepsis.

**Method:**

Patients who underwent salvage surgery for pelvic sepsis after prior low anterior resection or Hartmann's procedure for rectal cancer were prospectively maintained in a database between 2010 and 2020 and reviewed retrospectively. The primary endpoint was the incidence of IUI. Secondary endpoints were timing of diagnosis (intra‐ vs. postoperative), reinterventions related to the IUI and healing of IUI.

**Results:**

In total 126 consecutive patients were included, and IUI occurred in 13 patients (10%). A ureteric injury occurred in eight patients, bladder injury in four patients and a urethral injury in one patient. All patients with an IUI had radiotherapy as neoadjuvant treatment. The IUI was diagnosed postoperatively in 63% (*n* = 8/13) with a median duration between surgery and diagnosis of the IUI of 10 days (IQR: 6–15). The median number of reinterventions was five (range 1–31) in the group with a postoperative diagnosis and one (range 0–1) in the group with an intraoperative diagnosis. Four patients required a surgical reintervention, all concerning injuries diagnosed postoperatively. At the end of follow‐up, 85% of patients (*n* = 11/13) had a healed IUI.

**Conclusion:**

Iatrogenic urinary tract injury is not uncommon in salvage procedures for pelvic sepsis, even in an experienced tertiary referral centre. Most injuries were diagnosed postoperatively which affects the severity of these complications, emphasising the need to improve intraoperative diagnostic modalities.

**What does this paper add to literature**

Iatrogenic urinary tract injuries (IUI) during salvage surgery for pelvic sepsis are associated with high morbidity depending on the location and timing of diagnosis. This is the first large observational study on this topic, which demonstrates the relatively high incidence, the often late diagnosis, and the frequent need for reinterventions.

## INTRODUCTION

Pelvic sepsis may develop if an anastomotic leakage (AL) persists after a low anterior resection (LAR) or in case of blow‐out of the rectal stump after low Hartmann's procedure [1]. This is a complex problem as patients with pelvic sepsis are often subject to multiple interventions (endoscopic, radiological and surgical) to obtain local control. Salvage procedures for pelvic sepsis are a surgical challenge due to inflammatory‐, radiation‐ and surgically induced fibrotic scarring and altered anatomical planes, and associated with high perioperative morbidity rates [2]. In particular, such salvage procedures for pelvic sepsis might be prone to iatrogenic urinary tract injuries (IUI) [3].

Iatrogenic urinary tract injuries occur in approximately 0.3%–1.7% of elective colorectal surgeries. Despite the relatively low incidence, IUI are still considered a dreadful complication [3, 4]. These injuries are associated with significant morbidity due to several risks including urinoma and fistula formation and urinary tract infection with possible loss of renal function. The outcomes depend on location of the injury and timing of diagnosis [4, 5]. Often, IUIs are recognised postoperatively and may require a temporary diverting nephrostomy and secondary surgery at a later stage [6].

Data on the occurrence of IUI after redo abdominal and pelvic surgery, and in particular salvage management for pelvic sepsis is lacking. We hypothesized that major salvage surgery for pelvic sepsis is associated with a higher incidence of IUI with more often postoperative diagnosis related to the difficult intraoperative identification in the irradiated, inflamed and fibrotic operative field. The aim of this single centre, retrospective study was to evaluate the incidence, consequences and healing of IUI in major salvage surgery for pelvic sepsis after a LAR or Hartmann's procedure.

## MATERIALS AND METHODS

### Patients

All consecutive patients undergoing major salvage surgery between January 2010 and January 2020 after prior LAR or Hartmann's procedure for rectal cancer were included. These patients were treated for acute or chronic pelvic sepsis at a single tertiary referral centre (the Amsterdam University Medical Centres [AUMC] – location AMC). Pelvic sepsis was defined as uncontrolled persisting inflammation within the pelvic cavity following AL, including leakage from a rectal remnant after primary low Hartmann's procedure or dismantled anastomosis. Pelvic sepsis was diagnosed during physical examination, endoscopy, radiological imaging, or a combination of diagnostic modalities, and defined as chronic when sepsis was still present 12 months following the index procedure. Major salvage surgery included fistula excision, omentoplasty, muscle‐ or fasciocutaneous flap, redo coloanal anastomosis, end colostomy with takedown of the anastomosis and intersphincteric resection of rectal stump.

The primary endpoint was the incidence of IUI. Secondary outcomes included timing of diagnosis of the injury (intra‐ vs. postoperative), diagnostic modality, injury related reinterventions and percentage of patients with healed IUI. IUIs were considered to be healed if there were no clinical symptoms related to the urinary tract and continuity with unimpeded flow based on contrast imaging.

The study was approved by the medical ethical committee of the AUMC – location AMC (reference number W21_099 # 21.112).

### Data collection

Patient and treatment characteristics were retrospectively collected from medical charts and stored in an electronic database. Preoperative patient demographics, body mass index, medical and surgical history, and radiation therapy were reviewed. Operative records were reviewed for placement of preoperative ureteral stents, operating time, operative technique, and extent of intraoperative adhesions. IUIs were reviewed for location of injury, timing of diagnosis, clinical presentation, diagnostic modality, and management following Clavien Dindo classification [7].

### Statistical analysis

Categorical data were compared using the Chi‐Squared test, or the Fisher exact test when appropriate, and were presented as numbers and proportions. Numerical data were compared using the independent *t* test or Man‐Whitney U test according to distribution. The outcomes were reported as means with standard deviation (SD) or medians with interquartile range (IQR). The median number of reinterventions per patient were reported with IQR, as well as the range to represent interindividual variability. The statistical significance level was set at a *p*‐value of < 0.05. IBM SPSS Statistics for Windows (v.26.0, IBM Corp.) was used for the statistical analyses.

## RESULTS

### Baseline characteristics

In total, 126 consecutive patients underwent salvage surgery for pelvic sepsis, of whom 73% (*n* = 92/126) were male. Mean age was 62.5 years (± SD 11). The index procedure for rectal cancer consisted of a LAR in 91% (*n* = 115/126) of the patients and 94% (*n* = 118/126) of patients had undergone neoadjuvant (chemo) radiotherapy. At our centre, 82 patients (65%) underwent major pelvic surgical interventions related to leakage of the anastomosis or rectal remnant prior to salvage surgery, ranging between one and 28 interventions (median 1, IQR: 1–2), with 44 patients (35%) who underwent nonsurgical interventions (range 0–5, median 0, IQR: 0–1). The pelvic anatomy at the time of salvage surgery and other baseline characteristics are outlined in Table 1. Median time between index procedure and salvage surgery was 26 months (IQR: 14–65).

**TABLE 1:** Baseline characteristics

	*n* = 126
Sex, male	92/126 (73)
Age at time of salvage, mean ± SD (years)	62.5 ± 11.0
BMI, mean ± SD (kg/m^2^)	25.6 ± 3.6
ASA classification	
ASA I	25/126 (20)
ASA II	76/126 (60)
ASA III	23/126 (18)
ASA IV	2/126 (2)
Active smoker	20/126 (16)
Diabetes mellitus type II	20/126 (16)
Neoadjuvant therapy	
None	8/126 (6)
Short‐course radiotherapy only	59/126 (47)
Long‐course radiotherapy only	1/126 (1)
Radiotherapy only, type unknown	7/126 (6)
Short‐course radiotherapy followed by chemotherapy	5/126 (4)
Chemoradiotherapy	46/126 (37)
Index surgery	
Low anterior resection	115/126 (91)
Hartmann procedure	11/126 (9)
Pelvic status before salvage	
Rectal extirpation	3/126 (2)
Anastomosis in situ	92/126 (73)
Primary Hartmann	10/126 (8)
Secondary Hartmann	21/126 (17)
Time between salvage surgery and last date of follow‐up, months, median (IQR)	48 (23–72)

*Note:* Descriptive statistics are presented in proportions, unless otherwise stated.

Abbreviations: ASA, American Society of Anaesthesiology; BMI, body mass index; IQR, interquartile range; SD, standard deviation.

Prior to the salvage surgery, 4% (*n* = 5/126) of the patients had a urinary complication. Three patients had stenosis of the ureter, two requiring endoscopic stent placement and the other patient underwent a radiological nephrostomy placement on five occasions. Two patients had a fistula from the urethra to the colon and rectum, one of them resulting in a bladderneck stenosis, requiring an endoscopic bladder neck incision.

### Intraoperative characteristics

In two patients (1.5%), a prophylactic ureteral stent was inserted pre‐operatively. Both these patients had a history of urinary complications before salvage treatment in our institution: both had unilateral ureteric obstruction related to pelvic sepsis, with progressive hydronephrosis in one of these patients. A suprapubic catheter was inserted uncomplicatedly intraoperatively in 65% of the patients (*n* = 82/126), 78% (*n* = 64/82) of them were male. Of the 11 patients with a Hartmann's procedure as index procedure, (74%) eight underwent an intersphincteric resection of the rectal stump and the three remaining patients had restoration of continuity as salvage surgery. Of 115 patients with a LAR as index procedure, redo coloanal anastomosis was performed in 47% (*n* = 54/115) of patients, intersphincteric resection of the rectal stump after prior take down of the anastomosis in 17% (*n* = 21/115), and intersphincteric resection of the leaking anastomosis and creation of and end colostomy in 35% (*n* = 40/115).

In most patients (94%, *n* = 119/126), a combined abdominoperineal approach was pursued, while an isolated abdominal or perineal approach was used in two (2%) and five (4%) patients, respectively. For the abdominal part of the procedure, a minimally invasive approach by laparoscopy was performed in 58% of cases (*n* = 70/120), and by TAMIS in 56% (*n* = 70/124) for the perineal approach. Conversion from laparoscopy to a midline laparotomy was necessary in three patients (4%) due to dense adhesions. Extensive adhesiolysis was necessary in 36% (*n* = 45/126) of the patients. The ureters were identified intraoperatively in 29% (*n* = 36/126) of the patients with ureterolysis in 22 patients. Median operating time was 373 min (IQR: 297–500) in the group with an IUI, compared to 292 min (IQR: 241–342) in the group without an IUI. There were no other intraoperative complications.

### Iatrogenic urinary tract injuries

An IUI occurred during salvage surgery in 13 patients (10%). An IUI did not occur during salvage surgery in any of the patients with a prior urological complication. All patients with an IUI had radiotherapy as neoadjuvant treatment. Two out of 13 patients (15.4%) were smokers. Of the 13 patients with an IUI, a unilateral ureteric injury occurred in seven patients, bilateral ureteric injury in one patient, bladder injury in four patients and a urethral injury in one patient. The damage to either ureter, bladder or urethra was noticed intraoperatively in five patients. The diagnostic modality used to detect IUI postoperatively in the other eight patients is specified in Table 2. In all patients with ureteric injuries, a ureterolysis was performed intraoperatively, and resulted in direct damage to the ureter in three patients (patients 6, 9, 13). These injuries were recognised intraoperatively, and required reinsertion of the ureter in two patients, while one injury could be repaired by primary suture. The other five iatrogenic ureter injuries were picked up between postoperative day six to postoperative day 18 (patient 1, 5, 10–12), and were either a missed injury or secondary to ischaemia following the ureterolysis. One entry into the bladder was recognised intraoperatively and was repaired by a primary two‐layer closure. In one patient (patient 3) a urethral injury occurred, that was immediately closed over a transurethral catheter. Median duration between surgery and diagnosis of the IUI in patients with postoperative detection was 10 days (IQR: 6–15).

**TABLE 2:** Characteristics of patients with iatrogenic urinary tract injury

Patient	Year salvage	Type of injury	Procedure	Abdominal approach	Perineal approach	Time of diagnosis	Symptoms	Diagnostic modality	Initial management
1.	2015	Distal left ureter	Redo anastomosis	Open	Open	POD 6	Fever, abdominal pain	Elevated creatinine in fluid	‐
2.	2014	Bladder	Take down anastomosis, end colostomy	Open	Open	POD 4	Leakage of urine from wound	CT IVP	‐
3.	2020	Urethral	Redo anastomosis	Open	TAMIS	Intraoperatively	n/a	Gel leakage through TUC	Primary repair over urethral catheter
4.	2014	Bladder	Intersphincteric completion proctectomy with omentoplasty	Open	Open	POD 15	Leakage of urine from anal wound	CT scan	‐
5.	2016	Distal left ureter	Interpshincteric resection rectal stump	Open	Open	POD 12	Abdominal pain, pyrexia	CT IVP	‐
6.	2017	Distal left ureter	Intersphincteric completion proctectomy	Laparoscopic	TAMIS	Intraoperatively	n/a	Identification ureter	Reinsertion ureter by psoas hitch
7.	2019	Bladder‐ urethra transition	Intersphincteric resection rectal stump	Open	TAMIS	POD 8	Fever, increased drain output	CT scan	‐
8.	2019	Bladder, trigonum	Intersphincteric resection rectal stump	Open	Open	Intraoperatively	n/a	Entry to bladder	Primary repair and SPC
9.	2014	Distal right ureter	Redo anastomosis	Open	Open	Intraoperatively	n/a	Identification ureter	Reinsertion ureter by oversuturing over stent
10.	2016	Distal right ureter	Redo anastomosis	Laparoscopic	TAMIS	POD 18	Abdominal pain, leakage of urine from anal wound	CT scan	‐
11.	2016	Bilateral ureter	Redo anastomosis	Laparoscopic	TAMIS	POD 13	Urinary retention	CT scan	‐
12.	2018	Distal left ureter	Redo anastomosis	Laparoscopic	TAMIS	POD 6	Urinary retention	CT scan	‐
13.	2011	Middle right ureter	Intersphincteric resection rectal stump	Open	Open	Intraoperatively	n/a	Identification ureter	Primary repair

Abbrevations: CT IVP, CT intravenous pyelography; n/a, not applicable; POD, postoperative day; SPC, suprapubic catheter; TAMIS: transanal minimally invasive surgery.

### Follow up iatrogenic injuries

Six patients did not require any further reintervention outside endoscopic removal of ureteric stents (patients 3, 6‐9, 13), and IUI was intraoperatively detected and managed in five patients (see Table 3).

**TABLE 3:** Reinterventions related to the IUI

Patient	Location injury	Moment of diagnosis	Reintervention			CD score	Healed EFU
			Radiological	Surgical	Endoscopic		
1.	Distal left ureter	Postoperatively	Nephrostomy tube (2)	Ileocystoplasty	Dilatation and stent placement	IIIb	Yes
2.	Bladder	Postoperatively	Nephrostomy tube (4)	‐		IIIa	Yes
3.	Urethral	Intraoperatively	‐	‐	‐	‐	Yes
4.	Bladder	Postoperatively	Nephrostomy tube (30)		Nephrostomy tube (1)	IIIa	No
5.	Distal left ureter	Postoperatively	Nephrostomy tube (2)	Reimplantation of the left ureter in psoas hitch	Stent placement (2)	IIIb	Yes
6.	Distal left ureter	Intraoperatively	‐	‐	Removement stent	IIIa	No
7.	Bladder‐ urethra transition	Postoperatively	‐	‐	‐	II	Yes
8.	Bladder, trigonum	Intraoperatively	‐	‐	‐	‐	Yes
9.	Distal right ureter	Intraoperatively	‐	‐	Removement stent	IIIa	Yes
10.	Distal right ureter	Postoperatively	Nephrostomy tube (7)	Ureteroplasty with Boari flap	Removement stent (1)	IIIb	Yes
11.	Bilateral ureter	Postoperatively	Nephrostomy tube (4)	Ileal interposition ureter	‐	IIIb	Yes
12.	Distal left ureter	Postoperatively	Nephrostomy tube (1)	‐	‐	IIIa	Yes
13.	Middle right ureter	Intraoperatively	‐	‐	‐	‐	Yes

*Note:* Number between brackets represents amount of performed procedures.

Abbrevations: CD, Clavien Dindo score; EFU, end of follow‐up; IUI, iatrogenic urinary tract injury.

Four patients with postoperatively‐detected ureteric injuries needed nephrostomy tubes as initial management (patients 5, 10–12), and subsequent ureteric reimplantation procedures in three patients; an ileal interposition for the patient with a bilateral ureteric injury, one psoas hitch and one Boari flap procedure. One patient had a stent placed after unsuccessful endoscopic dilatation of a ureteric stricture and eventually had a reconstruction by an ileal interposition (patient 1). Of patients with a bladder injury, two out of three patients with a postoperative diagnosis (patient 2, 4) required bilateral nephrostomy tubes to control urine leakage.

For all patients with IUI, the median number of overall reinterventions was one (range 0–31, IQR: 1–5). Patients with a postoperative diagnosis of the IUI had a median number of overall reinterventions of five (range 1–31) and patients with intraoperative diagnosis had a median of one reintervention (range 0–1). All radiological and surgical reinterventions were performed in patients with postoperative detection of the injury. Readmission related to the IUI which occurred in 46% (*n* = 6/13), with a median admission length of 11 days (range 2–13).

At the end of follow‐up, 85% of patients (*n* = 11/13) had a healed IUI. The median duration from the occurrence of the IUI until healing was 8 months (IQR: 1–16). Patients with intraoperative diagnosis had a median time to healing of 1 month (IQR: 1–9), whereas patients with postoperative diagnosis had a median time to healing of 8 months (IQR: 7–60). There were two patients (patient 4 and 6) with persisting problems of IUI; one (patient 4) still has nephrostomy tubes in situ due to stenosis of the ureters without reconstruction options with a healed injury of the bladder on radiological imaging, and the other patient (patient 6) has persistent obstruction symptoms without obstruction identified by imaging. There was no mortality associated with the IUI. None of the IUI led to chronic kidney problems.

## DISCUSSION AND CONCLUSIONS

The present study reveals that IUI occurs in 10% of the patients undergoing salvage surgery for pelvic sepsis after prior LAR or Hartmann's procedure for rectal cancer. All patients with IUI had neoadjuvant radiotherapy before surgery. The majority were diagnosed postoperatively with a median delay of 10 days with subsequent need for radiological and surgical reinterventions, while intraoperatively detected injuries did not require such reinterventions. Eventually, most patients did not have long‐term sequelae related to the IUI.

In the current literature, IUI are portrayed as a rare complication, but this scarcely available data only reflects primary, elective surgery [3, 4, 6, 8]. So far, no studies have reported the incidence during complex abdominal or pelvic redo surgery. The substantially higher incidence after redo surgery compared to primary colorectal surgery is probably explained by a combination of factors that complicate the pelvic dissection, such as extensive scar tissue related to radiotherapy, previous surgery and chronic inflammation, as well as altered anatomy.

In nearly two thirds of patients the injury was recognised postoperatively. This is in line with prior studies which report that 50%–70% of IUI are identified postoperatively [9, 10]. Similar to other studies, these patients typically present with flank or abdominal pain, fever, ileus and/or urinary discharge via the anal canal, perineal wound or pelvic drain. The diagnosis of IUI, if not recognised intraoperatively, is usually several days postoperatively, although reports on timing are inconsistent [8, 11] [12]. We found that IUIs were diagnosed after a median period of 10 days in this setting of salvage surgery (IQR: 6–15). The relatively long interval suggests that the pathophysiological mechanism of postoperatively detected leakage might often be ischaemia, rather than a full thickness injury with direct urinary leakage. Perfusion of the ureters might already have been compromised by previous radiotherapy, and the use of diathermy and sealing devices during salvage surgery might result in secondary necrosis.

As expected, we detected a clear difference in morbidity between IUI detected during or after surgery in favour of the former group, as reflected by the median number of reintervention and the type of reinterventions. Intraoperative identification of the urological structures in close proximity to the field of dissection is essential to prevent injury. Furthermore, intraoperative detection of potential injury is key to immediate repair and is known to result in better long‐term outcomes [12]. However, identification of the ureter is most often achieved by visual inspection and palpation, which can both be challenging during minimal invasive surgery, especially in this specific patient population with extensive fibrosis. Furthermore, posterior displacement of pelvic organs typically occurs after primary or secondary Hartmann's procedures. This will hamper correct identification of the distal part of the ureters and vesicoureteric junctions. If restoration of continuity is aimed for during salvage surgery, dissection of the bladder and ureters might be necessary to create enough space for the colon to reach the rectal remnant. To minimise the risk of IUI involvement of an experienced surgeon is important with pre‐emptive or reactive consultations with an urologist if there are any concerns intraoperatively. In addition, in this setting of redo pelvic surgery for pelvic sepsis, there is a need for techniques to improve the visualisation of the urinary tract.

Prophylactic ureteral stenting (PUS) has gained popularity in the last couple of years with the purpose of preventing ureteral injuries [13]. However, no guidelines support its efficacy, as most studies show no benefit in the use of PUS in the incidence of IUI [14, 15]. By way of contrast, the use of PUS itself is demonstrated to be associated with high rates of iatrogenic urinary tract injury [16]. Even though there might not be a role for PUS to prevent IUI in primary surgery, it can still be a helpful tool to visualise the urological tract during redo surgery and ensure early recognition of IUI. However, one must be aware that ureteral stents are not always palpable in fibrotic tissue and do not prevent bladder and urethral injuries, neither do they influence the risk of ischaemic perforations with late urinary leakage.

A potential safer alternative for intraoperative visualisation of the entire urological tract (not limited to the ureters) is near‐infrared fluorescence imaging. Proof of concept studies suggest a promising future, being able to identify 22% (*n* = 14/62) of ureters that were not visible in white light [17]. Near‐infrared fluorescence showed the ureter to be in a different location than expected in 16% (*n* = 10/62) of the cases. From currently available fluorescent dyes, methylene blue is the best option that is readily available for fluorescence imaging of the ureter owing to its simple method of administration (intravenously) and efficacy [18]. However the main limitation of methylene blue is the low excitation coefficient, which may hamper visualisation of the urological tract in fibrotic tissue. An experimental near infrared agent, ZW800‐1, is promising for ureter detection in the future [19]. Phase I–II studies show a good safety profile of ZW800‐1 in patients with normal renal function, and detection of ureters is possible with low dosages. A further advantage of ZW800‐1 is that its excitation and emission spectrum overlaps with that of indocyanine green, a clinically available and frequently used fluorescent agent. ZW800‐1 can therefore be visualised with the same imaging systems at 800 nm. Visualisation of the urological tract via near‐infrared fluorescence imaging, may also prevent the need for ureterolysis, which in our series led to direct injury in three patients and devascularisation with delayed stenosis or leakage in five patients.

A substantial limitation of this study is its retrospective design. Patients were prospectively included, but specific data on IUI were retrieved retrospectively. For this reason, there were no established moments for measuring renal function (i.e., creatinine). In addition, the study was limited due to the low absolute number of events of IUI, which prevented the detection of independent predictors.

In conclusion, this is the first study presenting the incidence of IUI in a large cohort of patients undergoing redo pelvic surgery for pelvic sepsis with often prior radiotherapy. Results from this study support that IUI is a more frequent complication during salvage surgery with an incidence of 10%, as compared to primary colorectal resection. With regard to implications for clinical practice, our findings demonstrate that awareness of potential IUI is warranted during this type of surgery and patients can be counselled on the expected course and outcome. In addition, our findings outline the need for other intraoperative diagnostic modalities to assist in recognition of the urological tract in this complex patient group. PUS might help visualise the ureter, however fluorescence imaging might have the most value as it has the potential to visualise the whole urinary tract.

## CONFLICT OF INTEREST

The authors declare that they have no conflict of interest.

## AUTHOR CONTRIBUTIONS

All authors made substantial contribution to the study and the manuscript. All authors gave final approval of the version of the manuscript to be published. Johanna J. Joosten contributed in study design, acquisition of data, data analysis, interpretation of the data and participated in drafting of the article. Sarah Sharabiany contributed in study design, acquisition of data, data analysis, interpretation ofthe data and revised the article critically. Gijsbert D. Musters contributed in study design, interpretation ofthe data and revised the article critically. Harry P. Beerlage contributed in study design, interpretation ofthe data and revised the article critically. Pieter J. Tanis contributed in study design, interpretation ofthe data and revised the article critically. Wilhelmus A. Bemelman contributed in study design, interpretation ofthe data and revised the article critically. Roel Hompes contributed in study design, data analysis, interpretation of the data and participated in drafting of the article and final revision of the article.

## ETHICAL APPROVAL

This study has been approved by the medical ethical committee of the UMC – location AMC and has therefore been performed in accordance with the ethical standards laid down in the 1964 Declaration of Helsinki and its later amendments.

## Data Availability

The data that support the findings of this study are available on request from the corresponding author. The data are not publicly available due to privacy or ethical restrictions.
